# Updating the distribution of sand flies in Hungary with implications on their biology and ecology

**DOI:** 10.1016/j.crpvbd.2025.100293

**Published:** 2025-07-08

**Authors:** Katharina Platzgummer, Edwin Kniha, Vít Dvorak, Petr Halada, Julia Walochnik, Barbora Vomackova Kykalova, Ida Hanusniakova, Robert Farkas, Petr Volf, Attila J. Trájer

**Affiliations:** a Institute of Specific Prophylaxis and Tropical Medicine, Center for Pathophysiology, Infectiology and Immunology, Medical University Vienna, Vienna, Austria; b Department of Parasitology, Faculty of Science, Charles University Prague, Prague, Czech Republic; c BioCeV, Institute of Microbiology of the Czech Academy of Sciences, Vestec, Czech Republic; d Department of Parasitology and Zoology, University of Veterinary Medicine, Budapest, Hungary; e University of Pannonia, Sustainability Solutions Research Lab, Veszprém, Hungary

**Keywords:** Central Europe, Sand flies, MALDI-MS, Barcoding, Blood meal, Ecology

## Abstract

In Europe, sand flies (Diptera: Psychodidae: Phlebotominae) are characteristic Mediterranean fauna, though some species expand their range further north. However, the sand fly fauna of Central Europe remains underreported, particularly in Hungary where recent data is lacking due to limited and outdated entomological surveys. To address this gap, a series of sand fly surveys were conducted in Hungary, with significant findings from two trapping efforts in 2017 and 2024. In 2017, only a single female *Phlebotomus papatasi* was trapped in northern Hungary, which marks one of the northernmost records of the species. In 2024, a more extensive and geographically wider survey recorded 264 sand flies at 34 sites, including three species: *Ph. mascittii*, *Ph. neglectus*, and *Ph. papatasi*. Sand flies were found across diverse environmental settings, including urban, agricultural, and natural habitats. Particularly, the previously rare presence of *Ph. mascittii* at rural sites (natural rock formations) was reported. Analysis of historical and current data revealed the presence of four sand fly species in Central and South Transdanubia, with evidence suggesting potential range expansion. Blood meal analysis of engorged females identified a variety of domestic and wild host species, but no *Leishmania* or *Phlebovirus* infections were detected. Habitat modelling and linear discriminant analysis indicated substantial climate suitability across Southeast Europe, with most positive sand fly observations observed in discontinuous urban fabric CORINE Land Cover classes. This study offers important insights into the ecology, distribution, and climatic preferences of sand flies in Hungary and provides crucial baseline data to monitor potential future spread.

## Highlights

• *Phlebotomus mascittii*, *Ph. neglectus*, and *Ph. papatasi* were identified in the field surveys in Hungary.
• Co-occurrence of species was observed at several sites in habitats, including rock formations and private properties.
• Blood meal analysis revealed diverse hosts, including both domestic and wild animals, but no pathogens were detected.
• Habitat modelling and linear discriminant analysis indicated substantial climate suitability across Southeast Europe.

## Graphical abstract

**Figure:**
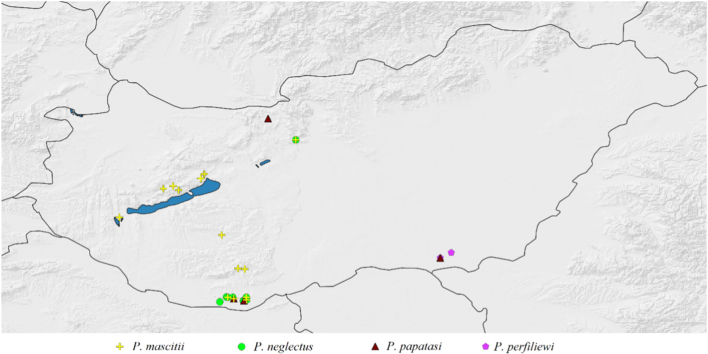

## 1 Introduction

Sand flies (Diptera: Psychodidae: Phlebotominae) are of medical relevance due to their role as vectors of a wide array of pathogens (Maroli et al., 2013; Ready, 2013). About 100 of the approximately 1000 described species impact global public health, particularly by transmitting the protozoan parasites *Leishmania* spp., which are the causative agents of human and animal leishmaniasis, one of the world’s most neglected tropical diseases. Sand flies can also transmit phleboviruses and the alpha-proteobacterium *Bartonella bacilliformis*, the causative agent of Carrion’s disease (Minnick et al., 2014; Akhoundi et al., 2016). Sand flies thrive in diverse biomes, from tropical rainforests and dry savannahs to temperate woodlands and peri-urban environments (Cecílio et al., 2022). Furthermore, they display a remarkable adaptability to both natural and anthropogenic habitats (Alten et al., 2016). Due to this ecological plasticity, combined with their wide geographic range, sand fly-borne diseases are endemic in at least 98 countries including North and East Africa, the Mediterranean Basin, the Middle East, South and Central Asia, and the Americas, accounting for more than one billion people living in areas at risk (WHO, 2021).

The Balkans and Southeast Europe are an important transitional zone between the leishmaniasis-endemic regions of the Middle East and the non-endemic regions of Europe. Due to the complex geography, heterogenous climate, and turbulent political evolvement, this region has become a focus of European sand fly studies only recently. It represents both a historically endemic but understudied area for leishmaniasis and a potential expansion front for both sand flies and sand fly-borne pathogens (Dvorak et al., 2020). This complex situation is influenced by climate change and other anthropogenic environmental alterations (Alten et al., 2016). Several *Phlebotomus* species occur across the Balkans and Southeast Europe, including proven vectors of *Leishmania* and phleboviruses, such as *Phlebotomus neglectus*, *Ph. perfiliewi*, *Ph. tobbi*, and *Ph. papatasi* (Cazan et al., 2019; Dvorak et al., 2020). Sand fly surveys in Bosnia and Herzegovina, Montenegro, Serbia, the Republic of Kosovo, Albania, and North Macedonia reported diverse sand fly assemblages, particularly in Mediterranean and sub-Mediterranean climate regions (Vaselek et al., 2017; Dvorak et al., 2020; Hoxha et al., 2024; Xhekaj et al., 2024). However, recent samplings identified sand flies also inhabiting cooler foothill and montane zones, a trend likely facilitated by climate change (Díaz-Sáez et al., 2021; Trájer, 2021).

*Phlebotomus neglectus* is considered the primary vector of *Leishmania infantum* in the Balkans, especially in coastal and lowland areas, while *Ph. perfiliewi* and *Ph. tobbi* contribute to rather local transmission cycles of the disease (Maroli et al., 2013; Xhekaj et al., 2023). Visceral leishmaniasis (VL), caused by *L. infantum*, has a long epidemiological history of endemicity in parts of Southeast Europe, particularly along the Adriatic coast, the Ionian region, and the Eastern Mediterranean (Vaselek, 2021a, 2021b). While the incidence of VL has declined in some countries, such as Greece, due to improved diagnostics, treatment, and vector control efforts, new autochthonous cases continue to be reported, particularly in rural and peri-urban areas where human-vector contact remains frequent (Antoniou et al., 2013) and where the parasite is circulating permanently in canine and feline populations as well as in wild canids among other mammals (Symeonidou et al., 2023). *Leishmania* spp. infections diagnosed in Central Europe are generally imported, primarily driven by human and dog travelling and migration. However, occasional autochthonous cases have also been recorded, suggesting that *Leishmania* spp. are on the verge (Kniha et al., 2023). Hungary is considered non-endemic for leishmaniasis; however, few records of imported and autochthonous cases in humans and animals deserve attention (Mihalca et al., 2019), particularly considering the endemicity of vector species such as *Ph. neglectus* and *Ph. perfiliewi* (Trájer, 2017).

One of the most concerning trends in Southeast Europe is the northward and altitudinal expansion of sand fly species, particularly *Ph. neglectus* and *Ph. perfiliewi*. This shift is partly driven by climate change, which is extending the suitable thermal niche for sand flies into previously cooler regions, along with land use changes that create favourable breeding and resting sites in peri-urban environments (Trájer et al., 2013; Alten et al., 2016). In northern Greece, for example, sand flies have been detected at elevations exceeding the 501–600 m above sea level range (Tsirigotakis et al., 2018). Although Hungary lies in the middle of the Carpathian Basin, right on the front line of the potential northward spread of sand flies, it represents a relatively blank spot on the map of Europe, as little relevant research has been conducted in the country.

## 2 Materials and methods

### 2.1 Entomological surveys and sampling regions

The first entomological survey was conducted in August 2–8, 2017, in the northeastern part of Hungary close to the Slovakian border, a presumed northern limit of sand fly distribution in the country. Sampling was conducted mostly indoor and outdoor, if feasible, at 31 peri-urban locations (always aiming for private properties with animals present) for one night at each location with one to six CDC miniature light traps baited with CO_2_ (John W. Hock Company, Gainesville, FL, USA) depending on the size of the property, resulting in a trapping effort of 86 trap-nights (Supplementary Table S1).

The second entomological survey was conducted in July 8–16, 2024, in the Balaton Valley and the southern part of Hungary close to the Croatian border at heterogenous trapping sites including 4 animal farms, 22 peri-urban (e.g. private households with animals), and 8 rural locations (rock formations and quarries). One or two CDC miniature light traps were either placed indoors or outdoors for one night at each location, resulting in overall 37 trap-nights (24 at peri-urban, 4 at agricultural, and 9 at rural locations) (Supplementary Table S1).

Informed verbal consent was obtained from all homeowners, and coordinates, locality type, trap position (indoor/outdoor), and animal presence were always recorded.

### 2.2 Morphological sand fly identification

Sand flies were dissected and heads and terminal segments of the abdomens were slide-mounted in CMCP-10 mountant (Polysciences, Inc., Warrington, PA, USA). Identification was based on descriptions of male genitalia, female spermathecae and pharyngeal armature in published morphological keys (Lewis, 1982; Dantas-Torres et al., 2014). The rest of the body parts were individually transferred to tubes and stored dry-frozen for molecular analyses.

The thoraces of 30 individuals caught in 2024 were separated from the rest of the bodies and stored at −80 °C for species identification by MALDI-TOF MS protein profiling. In addition, only the head was mounted from all engorged females and the remaining body stored at −80 °C for blood meal analysis using MALDI-FTICR MS peptide mapping and separate DNA extraction for blood-meal host identification by DNA sequencing.

### 2.3 Nucleic acid isolation

The trapped specimens were homogenized individually in 500 μl Dulbecco’s modified Eagle medium (DMEM) supplemented with 20% bovine serum albumin, 10 μg/ml gentamicin, 0.25 μg/ml amphotericin B and 1% penicillin/streptomycin (all from Gibco, Thermo Fischer Scientific, Waltham, MA, USA). Two metal beads (3 mm in diameter) were added to each 2.0-ml tube and the specimens were homogenized with a TissueLyser bead mill (Qiagen, Hilden, Germany), shaking at 50 Hz for 2 min. Centrifugation for 5 min at 8000 *rpm* was used to clear the homogenate in a tabletop centrifuge operating at 4 °C. For nucleic acid extraction, homogenates from up to five individuals were pooled by species and sex with a total volume of 200 μl homogenate per extraction. Parallel DNA and RNA extraction was performed using a DNA/RNA Micro kit 50 (Qiagen), with a final elution volume of 50 μl for DNA and 30 μl for RNA, following the manufacturer’s protocol with adjustments based on previous tests (Hoxha et al., 2024). The remaining homogenates were stored at −80 °C.

### 2.4 *cox*1 barcoding

For species-level identification, a standard DNA barcoding PCR was performed targeting a 658-bp fragment of the cytochrome *c* oxidase subunit 1 (*cox*1) gene with the primers LCO1490/HCO2198, in accordance with the previously described protocol (Folmer et al., 2014).

All PCR amplifications were performed in a final volume of 25 μl, using a 2× EmeraldAmp® GT PCR Master Mix (Takara Bio Europe AB, Göteborg, Sweden) with an Eppendorf Mastercycler (Eppendorf AG, Hamburg, Germany). The PCR products were analyzed using a Gel Doc™XR + Imager (Bio-Rad Laboratories Inc., Hercules, CA, USA), and the amplified fragments were purified using an Illustra™ GFX™ PCR DNA and Gel Purification Kit (GE Healthcare, Buckinghamshire, UK). The samples were sent to Microsynth (Microsynth Austria GmbH, Vienna, Austria) for Sanger sequencing, and the obtained bidirectional sequences were aligned with ClustalX 2.1 (Larkin et al., 2007) and edited with GeneDoc 2.7.0 (Nicholas and Nicholas, 1997). The generated consensus sequences were checked for stop codons, uploaded and compared to the minimum and maximum top 100 hits with > 86% query cover in the NCBI sequence database using BLAST.

### 2.5 Species identification by MALDI-TOF MS protein profiling

The thoraces were manually homogenized in 10 μl of 25% formic acid by a BioVortexer homogenizer (BioSpec, Bartlesville, USA) using disposable pestles for 15 s and centrifuged (10,000× *g* for 15 s). Two μl of the supernatant were mixed with 2 μl of MALDI matrix (sinapinic acid; 30 mg/ml in aqueous 60% acetonitrile/0.3% TFA), and 1 μl of this solution was deposited on a MALDI plate in duplicates. Protein profiles were acquired by an AutoFlex Speed MALDI-TOF spectrometer (Bruker Daltonics, Bremen, Germany) within a mass range of 3–25 kDa. For species identification, the profiles were processed by MALDI Biotyper v.3.1 (Bruker Daltonics) and searched against an in-house sand fly reference spectra database. Log score value (LSV) > 2.0 was considered a threshold for reliable species identification.

### 2.6 *Leishmania* spp. screening

Individual and pooled females were screened for *Leishmania* spp. in a probe-based qPCR protocol targeting the kinetoplast DNA (kDNA) using primers (F: 5′-CTT TTC TGG TCC TCC GGG TAG G-3′, R: 5′-CCA CCC GGC CCT ATT TTA CAC CAA-3′) and probe (FAM-TTTT CGC AGA ACG CCC CTA CCC GC-TAMRA) from Mary et al. (2004). The qPCR protocol offers a sensitive experimental detection of 10^1^ parasites (*L. infantum* and *L. major*) per ml, as shown by Prudhomme et al. (2024). In all reactions, negative (sterile H_2_O) and positive controls (*L. infantum* DNA corresponding to 10.2 parasites/ml) were used.

Quantitative PCR was conducted using a Luna® Universal Probe qPCR Master Mix (New England Biolabs, Ipswich, Massachusetts, USA) in a final volume of 20 μl with a Bio-Rad CFX96 Touch Real-Time PCR Detection System (Bio-Rad Laboratories, Inc., Hercules, CA, USA) with the following incubation program: (i) 95 °C for 3 min; (ii) 45 cycles with 1 cycle consisting of 95 °C for 15 s, 60 °C for 1 min.

### 2.7 *Phlebovirus* screening

All individuals and pools were screened for the presence of *Phlebovirus* RNA. First, a pan-*Phlebovirus* one-step RT-PCR protocol was applied using a SuperScript™ One-Step RT-PCR System with Platinum™ Taq DNA Polymerase (Thermo Fischer Scientific) with a final volume of 25 μl (Matsuno et al., 2015). The PCR products were analyzed using a Gel Doc™XR + Imager (Bio-Rad Laboratories Inc., Hercules, CA, USA). Additionally, two sensitive one-step RT-qPCR protocols were applied to screen for Toscana virus (TOSV, *Phlebovirus toscanaense*) (Pérez-Ruiz et al., 2007; Weidmann et al., 2008; Brisbarre et al., 2015) and sandfly fever Sicilian virus (SFSV; *Phlebovirus siciliaense*) (Alwassouf et al., 2016) using a Luna® Universal Probe One-Step RT-qPCR Kit (New England Biolabs, Ipswich, Massachusetts, USA). Negative (sterile H_2_O) and positive controls (for Pan-Phlebovirus RT-PCR: Punique virus (PUNV Tunisie2009 T101 strain), for TOSV RT-qPCR: Toscana virus (MRS2010 Marseille, 2010 strain), for SFSV RT-qPCR: Sandfly fever Sicilian virus (Italy, 1943 Sabin strain)) were used for all assays following an external quality assessment (EQA) (Ayhan et al., 2025). All primer sets and concentrations are given in Supplementary Table S2.

### 2.8 Blood meal analysis by PCR and sequencing

For blood meal analysis, a PCR was used to amplify a 16S rRNA gene fragment with the primers L2513/H2714 following the protocol of Kitano et al. (2007). In addition, the samples were subjected to an avian-specific PCR targeting a 220-bp fragment of the cytochrome *b* (*cytb*) gene using L15330AV (L0)/H15551AV (H1) primers as described in Lee et al. (2008). All PCRs were performed and analyzed as described above.

### 2.9 Blood meal analysis by peptide mass mapping MALDI-TOF mass spectrometry

The abdomen homogenization, trypsin digestion and sample preparation for MS analysis was performed as described (Hoxha et al., 2024). The peptide mass maps were measured on a SolariX XR 15T MALDI-FTICR mass spectrometer (Bruker Daltonics, Billerica, MA, USA) in the mass range of 300–6000 Da and then searched against UniProt database subset of vertebrate hemoglobin sequences using an in-house MASCOT 2.7 search engine (Matrix Science Inc., Boston, MA, USA) with the mass accuracy below 3 ppm.

### 2.10 CORINE Land Cover and Köppen-Geiger characterization

CORINE (Coordination of Information on the Environment) is a European programme initiated by the European Environment Agency (EEA) to collect and manage environmental data, with a particular focus on land cover. The CORINE Land Cover (CLC) dataset is one of the most widely used geospatial datasets for monitoring land use and land cover changes across Europe, and provides consistent, high-resolution land cover maps for Europe, updated every 6 years since 1990. It is based on satellite imagery and field surveys, offering a standardized classification system that enables cross-border environmental analysis. The CLC classification system consists of three hierarchical levels, with 44 detailed land cover classes at the third level.

The Köppen-Geiger system classifies climate into five main classes and 30 sub-types. The classification is based on threshold values and seasonality of monthly air temperature and precipitation (Beck et al., 2018).

All mappings of analyses were performed in QGIS version 3.34.11 using GRASS GIS version 8.4.0.

### 2.11 Linear discriminant analysis

Linear discriminant analysis (LDA) was used to compare the climatic needs of the four studied sand fly species. LDA is a supervised machine learning technique used for classification and dimensionality reduction. It is particularly useful when dealing with datasets containing multiple classes and seeks to maximize the separation between those classes. LDA aims to find a linear combination of features that best separates different classes. Unlike principal components analysis (PCA), which focuses on maximizing variance without considering class labels, LDA explicitly takes class information into account. The goal is to project high-dimensional data onto a lower-dimensional space while preserving class separability (Pedregosa et al., 2011). To perform the LDA analysis, 19 bioclimatic indicators (bio1 to bio19) were used (Supplementary Table S3). Linear discriminant analysis was implemented in Python version 3.10 (64-bit) using Anaconda 3 Navigator. To perform LDA, the “*sklearn*.*discriminant*_*analysis*” program library was used. Centroids and the Euclidean distances between them were calculated to assess class separation. To visualize the extent of species-specific climatic niches, convex hulls were drawn using the *ConvexHull* function from the “*scipy.spatial*” library.

## 3 Results

### 3.1 Sand fly trapping and identification

**Fig. 1:**
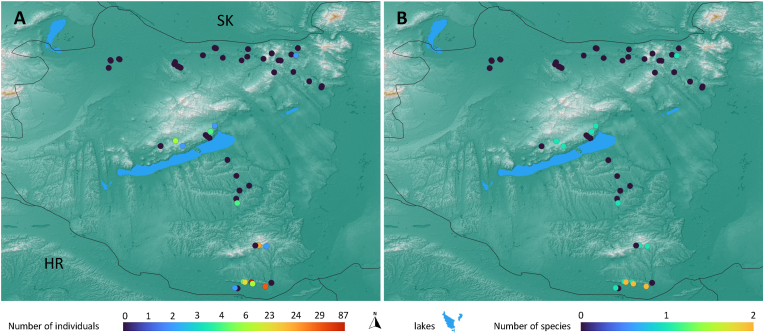
The number of trapped individuals (**A**) and the number of identified species (**B**).

In the first survey (year 2017), one female *Ph. papatasi* was caught in Epöl (northern Hungary), while all other trapping sites (*n* = 30, 96.8%) were negative. In the second survey (year 2024), 264 specimens were caught, comprising 96 (36.4%) males and 168 (63.6%) females (13 engorged). Three species were trapped, namely *Ph. mascittii*, *Ph. neglectus*, and *Ph. papatasi* (Table 1). Of 34 sampled sites, 16 (47.1%) were positive for sand flies (Fig. 1).

**Table 1:** Abundance of caught sand flies by species and sex in the 2024 survey in Hungary.

Species	Male	Female	Engorged	Total
*Ph. mascittii*	3 (6.7%)	42 (93.3%)	2	45
*Ph. neglectus*	90 (41.9%)	125 (58.1%)	11	215
*Ph. papatasi*	3 (75.0%)	1 (25.0%)	0	4
Total	96 (36.4%)	168 (63.6%)	13	264

**Fig. 2:**
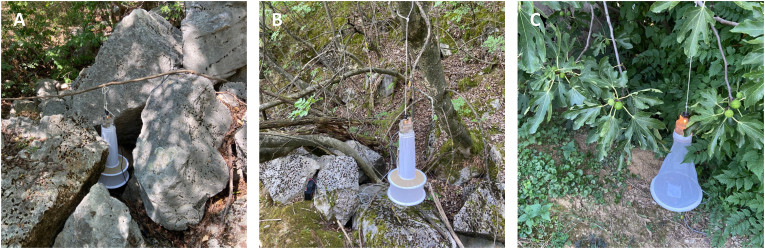
Placement of CDC light traps at rural trapping sites in Hungary. **A** Rock formation positive for *Ph. mascittii* and *Ph. neglectus*. **B** Rock formation in mixed forest positive for *Ph. neglectus*. **C** Fig tree close to stone house positive for *Ph. mascittii* and *Ph. papatasi*.

Generally, only one sand fly species was observed per location, except for four locations, where the presence of two sand fly species was noted (Supplementary Table S4). *Phlebotomus mascittii* and *Ph. neglectus* were co-occurring at a rock formation (Fig. 2A) and a quarry. *Phlebotomus neglectus* was generally highly abundant at rock formations and quarries (Fig. 2B); however, co-occurrence with *Ph. papatasi* was observed at a private property housing pigs and chickens. Co-occurring *Ph. mascittii* and *Ph. papatasi* were trapped under a fig tree close to a private property with stone houses (Fig. 2C).

### 3.2 Barcoding

In total, seven *cox*1 sequences with a length of 658 bp without primers were generated for all three detected species. All barcodes allowed identification to the species level. All three *Ph. neglectus* sequences (two haplotypes) showed the highest identity (99.85%) with a sequence originating from Serbia (GenBank: KY848830). The *Ph. papatasi* sequence also showed the highest identity (99.84%) with a sequence from Serbia (GenBank: KY848828), and *Ph. mascittii* sequences were 100% identical to sequences from Austria (GenBank: MN812830) (Table 2).

**Table 2:** Generated barcodes, haplotypes, and accession numbers of sand flies from Hungary.

Species	Barcodes	Haplotypes	GenBank ID	BLAST identity
Subgenus *Larroussius*				
*Ph. neglectus*	3	2	PV426659-PV426661	99.85% (KY848830)
Subgenus *Phlebotomus*				
*Ph. papatasi*	1	1	PV426662	99.84% (KY848828)
Subgenus *Transphlebotomus*				
*Ph. mascittii*	3	1	PV426656-PV426658	100% (MN812830)

### 3.3 MALDI-TOF MS protein profiling

The presence of the three sand fly species identified by *cox*1 barcoding was confirmed by MALDI-TOF protein profiling of 30 specimens (12 males, 18 females) caught in 2024. Except for one, all analyzed sand flies provided high-quality protein profiles, allowing reliable species assignment. Most of the samples were identified with LSV above 2.5, namely 14 *Ph. mascittii* (LSV 2.185–2.597), 11 *Ph. neglectus* (2.479–2.612), and 4 *Ph. papatasi* (2.483–2.563).

### 3.4 Sand fly distribution in Hungary

**Fig. 3:**
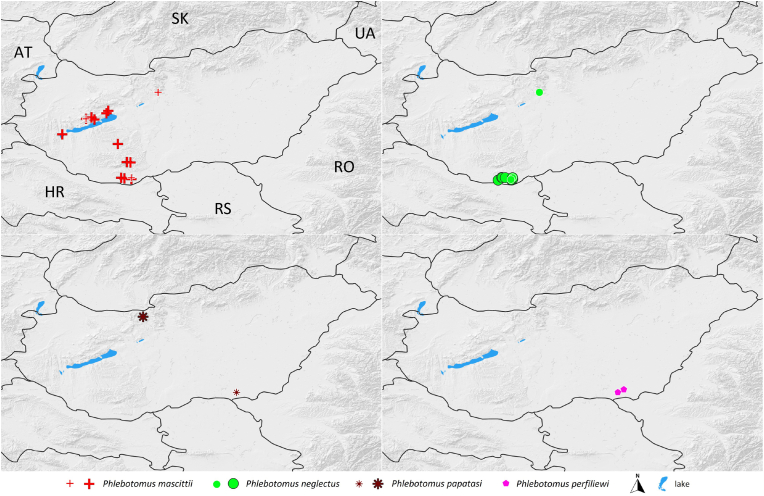
Distribution of four sand fly species in Hungary based on this study and historical data. Historical sand fly records are marked with white frames, recent records are marked with black framing.

Based on historical data (Farkas et al., 2011; Trájer, 2017) and our surveys presented here, four sand fly species have been recorded in Central and South Transdanubia in Hungary: *Ph. mascittii*, *Ph. neglectus*, *Ph. papatasi*, and *Ph. perfiliewi*. In the southern part of South Transdanubia, Baranya County, the presence of three species, namely *Ph. mascittii*, *Ph. neglectus*, and *Ph. perfiliewi*, has been reported, with two species occurring at one trapping site. The northernmost (47.6°) occurrence of a sand fly species, *Ph. papatasi*, was observed in Epöl, in Central Transdanubia, Komárom-Esztergom County, close to the Hungary-Slovakia border, representing one of the northernmost records of this species. In the Balaton Highlands, only *Ph. mascittii* was observed, in the close vicinity of the Balaton Riviera, in Litér and Szentkirályszabadja. This species was also trapped at the Little Balaton, which is the first observation of any sand fly species in this region. Similarly, *Ph. mascittii* was also found in Dalmand, which is the first observation of this species in the Dombóvár administrative region, in the central part of South Transdanubia (Fig. 3)

### 3.5 The altitudinal distribution

**Fig. 4:**
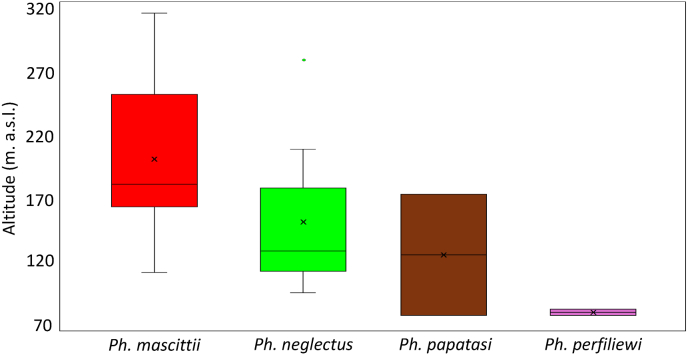
The altitudinal distribution of Hungarian sand fly occurrences by species.

The altitudinal range distribution of sand fly occurrences varies between species. The highest mean elevation of 206.3 m was found for *Ph. mascittii* (range: 118–316 m; SD: 57.8 m). The second highest mean elevation, 150.1 m, was observed for *Ph. neglectus* (range: 94–279 m; SD: 53.1 m). The mean elevation of *Ph. papatasi* was 137.4 m (range: 76–208 m; SD: 52.3 m). The lowest elevations were recorded for *Ph. perfiliewi* in Hungary with a mean elevation of 78.5 m (range: 76–81 m; SD: 3.5) (Fig. 4).

### 3.6 Blood meal analyses and pathogen screening

Altogether, we analyzed 13 engorged female sand flies of two species, namely two *Ph. mascittii* and eleven *Ph. neglectus*. Of these, 11 (84.6%) were successfully analyzed by combining MALDI-FTICR mass spectrometry and DNA sequencing, and five blood meal hosts were identified, including cat, dog, pig, deer, and chicken (Table 3). Ten single-origin blood meals and one mixed blood meal (chicken and pig) were found, of which eight were successfully identified by both techniques, including the mixed blood meal. Neither mass spectrometry nor DNA sequencing (16S-based) could discriminate between domestic pig (*Sus scrofa domesticus*) and wild boar (*S. scrofa*). At all private properties sampled, at least one of the resident animals was represented in the blood meal. At rural locations (rock formations and quarries), DNA of domestic and wild animals was observed (cat, roe deer, domestic pig/wild boar) (Table 3).

**Table 3:** Analyzed blood meals by MALDI-FTICR MS and sequencing. Chicken (*Gallus gallus*), cat (*Felis catus*), roe deer (*Capreolus capreolus*), dog (*Canis lupus familiaris*), and feral pig/wild boar (*Sus scrofa* dom.) were identified.

Species	Locality type	MALDI-FTICR MS	Sequencing	Host animals at site
*Ph. mascittii*	Private property	*G. gallus*	*G. gallus*	*G. gallus*, *C. lupus*
*Ph. mascittii*	Private property	–	–	*G. gallus*, *C. lupus*
*Ph. neglectus*	Rock formation	*S. scrofa* (dom.)	*S. scrofa* (dom.)	Unknown
*Ph. neglectus*	Rock formation	*C. capreolus*	*C. capreolus*	Unknown
*Ph. neglectus*	Quarry	*C. capreolus*	*C. capreolus*	Unknown
*Ph. neglectus*	Quarry	*F. catus*	*F. catus*	Unknown
*Ph. neglectus*	Quarry	–	–	Unknown
*Ph. neglectus*	Quarry	–	*C. capreolus*	Unknown
*Ph. neglectus*	Private property	*C. lupus*	–	*G. gallus*, *C. lupus*, *S. scrofa* (dom.)
*Ph. neglectus*	Private property	*S. scrofa* (dom.)	*S. scrofa* (dom.)	*G. gallus*, *C. lupus*, *S. scrofa* (dom.)
*Ph. neglectus*	Private property	*G. gallus*, *S. scrofa* (dom.)	*G. gallus*, *S. scrofa* (dom.)	*G. gallus*, *C. lupus*, *S. scrofa* (dom.)
*Ph. neglectus*	Private property	*G. gallus*	–	*G. gallus*, *C. lupus*, *S. scrofa* (dom.)
*Ph. neglectus*	Private property	*S. scrofa* (dom.)	*S. scrofa* (dom.)	*G. gallus*, *C. lupus*, *S. scrofa* (dom.)

Neither *Leishmania* DNA nor *Phlebovirus* RNA was detected in the samples.

### 3.7 CORINE Land Cover characterization

**Fig. 5:**
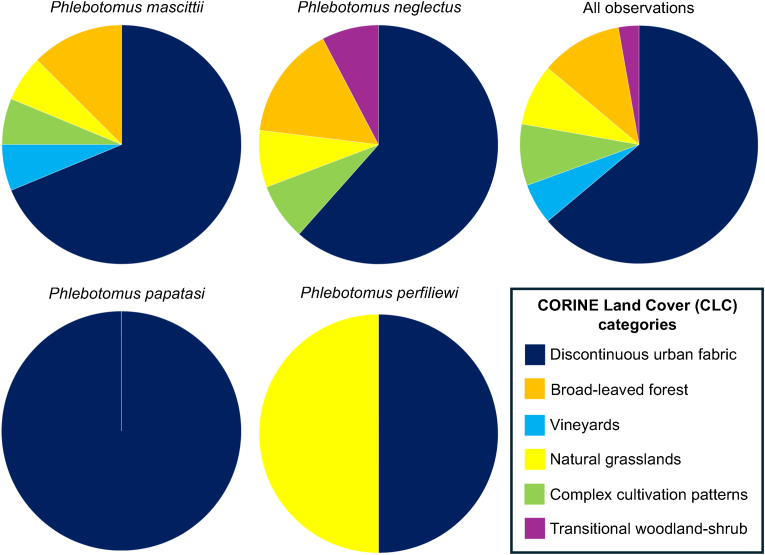
Pie-chart diagram of positive sand fly trapping observations (species × sites) in Hungary by CORINE Land Cover categories (CLCs).

Most of the positive sand fly observations (species × sites) were observed in discontinuous urban fabric CLCs (CLC 2; 22 observations; 66.7%). Others were natural grasslands (CLC 26; 4 sites; 12.1%), broad-leaved forest (CLC 23; 3 sites; 9.1%), and complex cultivation patterns (CLC 20; 2 sites; 6.1%). Positive trapping sites also included vineyards (CLC 15; 1 site; 3.0%) and transitional woodland-shrub (CLC 29; 1 site; 3.0%) (Fig. 5).

*Phlebotomus neglectus* was found in discontinuous urban fabric (CLC 2), complex cultivation patterns (CLC 20), broad-leaved forest (CLC 23), natural grasslands (CLC 26), and transitional woodland-shrub (CLC 29) land cover sites. *Phlebotomus mascittii* was found at all CLCs that were positive for sand flies in Hungary, except for transitional woodland-shrub (CLC 29). *Phlebotomus papatasi* was found only in discontinuous urban fabric (CLC 2) covered sites. *Phlebotomus perfiliewi* was caught in discontinuous urban fabric and broad-leaved forest sites (Fig. 5).

### 3.8 Köppen-Geiger characterization

**Fig. 6:**
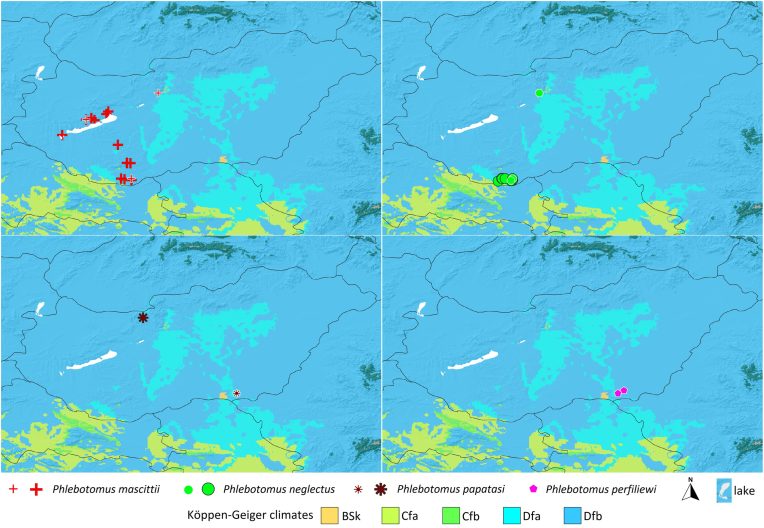
Köppen-Geiger categories within the potential range of the four sand fly species occurring in Hungary and bordering regions: *Ph. mascittii*, *Ph. neglectus*, *Ph. papatasi*, and *Ph. perfiliewi*. *Abbreviations*: BSk, cold semi-arid climates; Cfa, humid subtropical climate; Cfb, oceanic climate; Dfa, humid continental climate (hot summer subtype); Dfb, humid continental climate (warm summer subtype).

There was no notable difference between the Köppen-Geiger climate class preferences of the four studied sand fly species in Hungary. All four species were solely collected in warm summer continental (Dfb) sites (Fig. 6).

### 3.9 Linear discriminant analysis

**Fig. 7:**
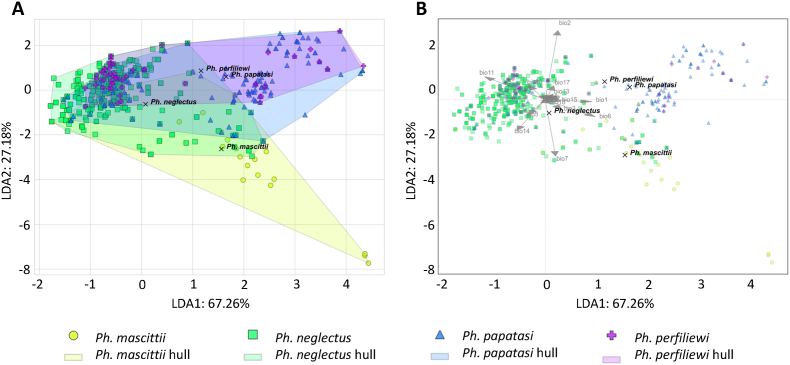
The results of linear discriminant analysis comparing climatic requirements of four sand fly species in the studied region with convex hulls (**A**) and biplots (**B**).

The linear discriminant analysis based on climatic variables showed that in Southeast Europe habitats of *Ph. papatasi* and *Ph. perfiliewi* were most similar to each other (Euclidean distance of the convex hulls was 0.56). The most notable distances were observed between *Ph. mascittii* and *Ph. perfiliewi* as well as between *Ph. mascittii* and *Ph. papatasi* (Euclidean distances of the convex hulls were 3.52 and 3.23, respectively) (Fig. 7A). The biplot results indicate that *Ph. mascittii* inhabits sites with high isothermality (bio3) and high minimum temperatures of coldest month values (bio6), while *Ph. papatasi* and *Ph. perfiliewi* prefer regions where the annual mean temperature (bio1) and precipitation seasonality (bio15) values are comparably high. In contrast, *Ph. neglectus* was observed to inhabit sites with balanced climatic conditions compared to the other three sand fly species (Fig. 7B).

## 4 Discussion

The results of two consecutive surveys on sand fly distribution in Hungary, conducted in 2017 and 2024, revealed previously unidentified sand fly presence and habitat preferences. These findings provide important insights into the ecological and biogeographical dynamics of these medically and veterinary important insects at the transition between Southeast and Central Europe. Our trapping activities, combined with historical data, expanded the known range of *Ph. papatasi* presence in Hungary to three regions. Similarly, the documented occurrence of *Ph. mascittii* was increased by two new regions. It was also proven that *Ph. mascittii* is present in a larger region of the Balaton highland than previously documented, and *Ph. mascittii*, *Ph. neglectus*, and *Ph. papatasi* all have a wide range on the southern slopes of the Villány Hills.

The co-occurrence of sand fly species at several sites in Hungary confirms that local sand fly populations can exploit a range of ecological niches, from domestic and peridomestic habitats at private properties to natural rock formations, as also observed in other studies (Alten et al., 2016; Veiga et al., 2024). A broad niche range is known for *Ph. papatasi*, which was shown to inhabit animal burrows (Wasserberg et al., 2003) as well as urban environments (Dantas-Torres et al., 2010), among others, and for *Ph. neglectus*, being highly abundant at breeding sites close to human dwellings, but also in caves (Xhekaj et al., 2024). However, *Ph. mascittii* presence is mostly reported at man-made habitats, e.g. animal barns, old sheds, garages or even cemeteries, which might be associated with a biased sampling effort at these locations (Kniha et al., 2020; Oerther et al., 2020; Bravo-Barriga et al., 2022; Hoxha et al., 2024). Here, we report the co-occurrence of *Ph. mascittii* and *Ph. neglectus* at a sylvatic rock formation urging to target sylvatic trapping sites with entomological surveys to identify the true distribution and habitat preferences of *Ph. mascittii*, which may be wider than currently known.

While we cannot correlate potential range expansion of sand flies in Hungary with climatic changes due to limited historical data available from the country, we observed a high diversity of CORINE Land Cover classes associated with positive sites, including discontinuous urban fabric, complex cultivation patterns, and areas with natural vegetation, which highlights the ecological flexibility of locally recorded species.

The predominance of *Ph. mascittii* in urban and peri-urban settings, the affiliation of *Ph. neglectus* to both agricultural and rocky habitats, as well as the frequent occurrence of *Ph. papatasi* in urbanized environments suggest that these species can exploit different ecological strategies (Wasserberg et al., 2003; Dantas-Torres et al., 2010; Kniha et al., 2020; Xhekaj et al., 2024). Moreover, the observation of *Ph. mascittii* at new northern sites such as the Balaton Highlands and Little Balaton supports the hypothesis that climate change, land use modifications and increasing human-mediated habitat connectivity may facilitate the northward expansion of sand fly species (Aspöck et al., 2008; Medlock et al., 2014).

The diversity of identified blood meal hosts further supports the notion that sand flies in Hungary are interacting with a variety of both domestic and wild animals, indicating their integration into diverse ecological networks. This ability to feed on multiple host species, including cats, dogs, pigs, deer, and chickens, further supports their adaptability to different landscapes, from private farms to natural habitats (Trájer and Kniha, 2025).

We observed a distinct difference in positive trapping sites between the 2017 and 2024 surveys, which can most plausibly be explained by geography for several reasons, with other confounding factors to be ruled out. Firstly, while the 2017 survey covered a similar number of trapping sites as well as similar peri-urban trapping locations, namely private properties with animals, the sampling effort (trap-nights) more than doubled in the 2024 survey. Secondly, during the 2017 survey, CDC light traps baited with CO_2_ were mostly deployed, known to increase trapping numbers for many Old-World species (Kasap et al., 2009), while only light trapping was performed during the 2024 survey. Thirdly, seasonality should not display a confounding factor as trappings were performed in early August and in early July in 2017 and 2024, respectively, which generally resembles peak activity of sand flies in temperate countries (Kniha et al., 2021). Thus, fourthly, geography provides the best explanation as a climatically less favoured area of northern Hungary has been surveyed in 2017, revealing only a single *Ph. papatasi* specimen, while the 2024 survey focused on a larger region in the southern part of the country, partially displaying features of Mediterranean climate, which favours sand fly presence and diversity. Additionally, the selection of more diverse trapping sites in 2024, including both anthropogenically influenced areas such as peri-urban gardens and livestock farms and semi-natural habitats such as rock formations and quarries, significantly improved the chances of detecting sand fly populations across a broader environmental gradient. Sand flies, being thermophilic species, are known to thrive in areas with mild winters and hot summers, which are increasingly characteristic of the Carpathian Basin due to climate change and might change sand fly communities in the future (Fischer et al., 2011; Trájer et al., 2013; Koch et al., 2017).

Noteworthy, the presence of *Ph. papatasi* and in parallel the observed absence of *Ph. mascittii* in the 2017 survey is quite surprising, as *Ph. mascittii* is generally the northernmost species found in Europe and considered to tolerate lower temperatures compared to other species (Kniha et al., 2021). However, *Ph. papatasi* was recently shown to have a high meteorological tolerance compared to other sand fly species present in Europe, which might promote survival or even spread to microclimatically suitable regions (Trájer and Kniha, 2025).

While we applied PCR protocols based on the highest molecular standards (Prudhomme et al., 2024; Ayhan et al., 2025), the absence of *Leishmania* DNA and *Phlebovirus* RNA in all tested specimens may be attributed to several factors. Of the potential vector species, only *Ph. neglectus*, the major vector for *L. infantum* in the Balkans (Velo et al., 2017; Xhekaj et al., 2023) and potential vector of recently described phleboviruses (Ayhan et al., 2016; Bino et al., 2019), was trapped in sufficient numbers. However, considering low infection rates among populations, the sample size was likely insufficient to enable detection of pathogens, which could be overcome with future longitudinal trappings, particularly at sites with vector species and sand fly-animal-human interaction.

While *Ph. papatasi* is the principal vector of *L. major* and Sandfly fever Sicilian virus (SFSV), only a few specimens of this species were caught. Moreover, the circulation of *L. major* in Central Europe is rather unlikely due to the absence of currently proven suitable reservoir hosts, which are gerbils (Antoniou et al., 2013). Noteworthy, *Ph. mascittii* is yet a suspected (Obwaller et al., 2016) but so far experimentally unproven vector of *L. infantum*.

Finally, the endemicity of leishmaniasis in Hungary is unclear. Animal leishmaniosis is not notifiable in Hungary, and the status of reporting human cases is unclear (Berriatua et al., 2021). Several imported cases of canine leishmaniosis have been reported (Farkas et al., 2011), and Tánczos et al. (2012) mention an unpublished account of dogs imported to Germany from Hungarian shelters tested positive for *Leishmania* by immunofluorescence antibody test. On the contrary, in a retrospective serological study of dogs living in Germany with travel history to Hungary all were tested negative (Hamel et al., 2011), and another retrospective study on dogs imported to Germany reported the absence of *Leishmania* DNA by PCR in dogs originating from Hungary (Röhrig et al., 2011); however, indirect and direct detection methods should generally be discussed separately. Noteworthy, no autochthonous but several imported human cases of visceral leishmaniasis, some with travel history to endemic countries (e.g. Croatia), have been reported in the last decades (Fried et al., 2003; Péterfi et al., 2011). Despite sporadic reports and potential underreporting, increasing import of dogs from Southeast Europe and the wide presence of *Ph. neglectus* in the country require continued surveillance.

## 5 Conclusions

This study provides new insights into the distribution and ecology of sand fly species in Hungary, showing new data on *Ph. mascittii*, *Ph. neglectus*, and *Ph. papatasi*. The findings from both field surveys highlight a yet underreported range of these species in Central and South Transdanubia, with *Ph. papatasi* marking one of its northernmost records in Epöl, Komárom-Esztergom County. Notably, the co-occurrence of multiple sand fly species at various trapping sites, particularly in rural environments such as rock formations and quarries, reveals complex habitat preferences and ability of sand flies to adapt to a range of ecological niches. These new findings indicate that sand fly populations in Hungary may be larger than previously reported and underscore the need for continued monitoring of sand fly populations, particularly considering their potential role as vectors of pathogens such as *Leishmania* and phleboviruses. Future studies should aim for larger sample sizes, testing over multiple seasons, and assessing vector competence to better inform public health strategies.

## CRediT authorship contribution statement

**Katharina Platzgummer:** Conceptualization, Formal analysis, Investigation, Methodology, Writing – original draft, Writing – review & editing. **Edwin Kniha:** Conceptualization, Investigation, Methodology, Project administration, Supervision, Writing – original draft, Writing – review & editing. **Vít Dvorak:** Methodology, Writing – review & editing. **Petr Halada:** Methodology, Investigation, Writing – review & editing. **Julia Walochnik:** Writing – review & editing. **Barbora Vomackova Kykalova:** Methodology, Writing – review & editing. **Ida Hanusniakova:** Methodology, Writing – review & editing. **Robert Farkas:** Methodology, Writing – review & editing. **Petr Volf:** Methodology, Writing – review & editing. **Attila J. Trájer:** Conceptualization, Formal analysis, Investigation, Methodology, Project administration, Writing – original draft, Writing – review & editing.

## Ethical approval

Not applicable.

## Funding

Funding provided by the RRF-2.3.1-21-2022-00014 National Multidisciplinary Laboratory for Climate Change. This paper was also supported by the János Bolyai Research Scholarship of the 10.13039/501100003825Hungarian Academy of Sciences under grant number: bo/00896/24/5. The study was partially funded by an OeAD WTZ Austria/Czech Republic project (CZ09/2024).

## Declaration of competing interests

The authors declare that they have no known competing financial interests or personal relationships that could have appeared to influence the work reported in this paper.

## Data Availability

All data generated or analyzed during this study are included in this published article and its supplementary files. The newly generated sequences were submitted to the GenBank database under the accession numbers PV426656-PV426662.

## References

[bib1] Akhoundi M., Kuhls K., Cannet A., Votýpka J., Marty P., Delaunay P., Sereno D. (2016). A Historical overview of the classification, evolution, and dispersion of *Leishmania* parasites and sandflies. PLoS Negl. Trop. Dis..

[bib2] Alten B., Maia C., Afonso M.O., Campino L., Jiménez M., González E. (2016). Seasonal dynamics of phlebotomine sand fly species proven vectors of Mediterranean leishmaniasis caused by *Leishmania infantum*. PLoS Negl. Trop. Dis..

[bib3] Alwassouf S., Christodoulou V., Bichaud L., Ntais P., Mazeris A., Antoniou M., Charrel R.N. (2016). Seroprevalence of sandfly‐borne phleboviruses belonging to three serocomplexes (Sandfly fever Naples, Sandfly fever Sicilian and Salehabad) in dogs from Greece and Cyprus using neutralization test. PLoS Negl. Trop. Dis..

[bib4] Antoniou M., Gramiccia M., Molina R., Dvořák V., Volf P. (2013). The role of indigenous phlebotomine sandflies and mammals in the spreading of leishmaniasis agents in the Mediterranean region. Euro Surveill..

[bib5] Aspöck H., Gerersdorfer T., Formayer H., Walochnik J. (2008). Sandflies and sandfly-borne infections of humans in Central Europe in the light of climate change. Wien. Klin. Wochenschr..

[bib6] Ayhan N., Baronti C., Thrion L., Bongiorno G., Maia C., Charrel R.N. (2025). External quality assessment for molecular detection of sand fly-borne phleboviruses circulating in the Mediterranean Basin. Parasites Vectors.

[bib7] Ayhan N., Velo E., de Lamballerie X., Kota M., Kadriaj P., Ozbel Y. (2016). Detection of *Leishmania infantum* and a novel *Phlebovirus* (Balkan virus) from sand flies in Albania. Vector Borne Zoonotic Dis..

[bib8] Beck H.E., Zimmermann N.E., McVicar T.R., Vergopolan N., Berg A., Wood E.F. (2018). Present and future Köppen-Geiger climate classification maps at 1-km resolution. Sci. Data.

[bib9] Berriatua E., Maia C., Conceição C., Özbel Y., Töz S., Baneth G. (2021). Leishmaniases in the European Union and neighboring countries. Emerg. Infect. Dis..

[bib10] Bino S., Velo E., Kadriaj P., Kota M., Moureau G., Lamballerie X. de (2019). Detection of a novel Phlebovirus (Drin virus) from sand flies in Albania. Viruses.

[bib11] Bravo-Barriga D., Ruiz-Arrondo I., Peña R.E., Lucientes J., Delacour-Estrella S. (2022). Phlebotomine sand flies (Diptera, Psychodidae) from Spain: An updated checklist and extended distributions. ZooKeys.

[bib12] Brisbarre N., Plumet S., Cotteaux-Lautard C., Emonet S.F., Pages F., Leparc-Goffart I. (2015). A rapid and specific real time RT-PCR assay for diagnosis of Toscana virus infection. J. Clin. Virol..

[bib13] Cazan C.D., Păstrav I.R., Ionică A.M., Oguz G., Erisoz Kasap O., Dvořák V. (2019). Updates on the distribution and diversity of sand flies (Diptera: Psychodidae) in Romania. Parasites Vectors.

[bib14] Cecílio P., Cordeiro-da-Silva A., Oliveira F. (2022). Sand flies: basic information on the vectors of leishmaniasis and their interactions with *Leishmania* parasites. Commun. Biol..

[bib15] Dantas-Torres F., Latrofa M., Otranto D. (2010). Occurrence and genetic variability of *Phlebotomus papatasi* in an urban area of southern Italy. Parasites Vectors.

[bib16] Dantas-Torres F., Tarallo V.D., Otranto D. (2014). Morphological keys for the identification of Italian phlebotomine sand flies (Diptera: Psychodidae: Phlebotominae). Parasites Vectors.

[bib17] Díaz-Sáez V., Corpas-López V., Merino-Espinosa G., Morillas-Mancilla M.J., Abattouy N., Martín-Sánchez J. (2021). Seasonal dynamics of phlebotomine sand flies and autochthonous transmission of *Leishmania infantum* in high-altitude ecosystems in southern Spain. Acta Trop..

[bib18] Dvorak V., Kasap O.E., Ivovic V., Mikov O., Stefanovska J., Martinkovic F. (2020). Sand flies (Diptera: Psychodidae) in eight Balkan countries: Historical review and region-wide entomological survey. Parasites Vectors.

[bib19] Farkas R., Tánczos B., Bongiorno G., Maroli M., Dereure J., Ready P.D. (2011). First surveys to investigate the presence of canine leishmaniasis and its phlebotomine vectors in Hungary. Vector Borne Zoonotic Dis..

[bib20] Fischer D., Thomas S.M., Beierkuhnlein C. (2011). Modelling climatic suitability and dispersal for disease vectors: The example of a phlebotomine sandfly in Europe. Procedia Environ. Sci..

[bib21] Folmer O., Black M., Hoeh W., Lutz R., Vrijenhoek R. (2014). DNA primers for amplification of mitochondrial cytochrome *c* oxidase subunit I from diverse metazoan invertebrates. Mol. Mar. Biol. Biotechnol..

[bib22] Fried K., Todorova R., Pintér E. (2003). Humán visceralis leishmaniosis megbetegedés Magyarországon. Epinfo.

[bib23] Hamel D., Röhrig E., Pfister K. (2011). Canine vector-borne disease in travelled dogs in Germany - a retrospective evaluation of laboratory data from the years 2004–2008. Vet. Parasitol..

[bib24] Hoxha I., Trájer A.J., Dvorak V., Halada P., Šupić J., Obwaller A.G. (2024). Phlebotomine sand flies (Diptera: Psychodidae) of Bosnia and Herzegovina: Distribution, ecology and environmental preferences. Acta Trop..

[bib25] Kasap O.E., Belen A., Kaynas S., Simsek F.M., Biler L., Ata N., Alten B. (2009). Activity patterns of sand fly (Diptera: Psychodidae) species and comparative performance of different traps in an endemic cutaneous leishmaniasis focus in Cukurova Plain, Southern Anatolia, Turkey. Acta Vet..

[bib26] Kitano T., Umetsu K., Tian W., Osawa M. (2007). Two universal primer sets for species identification among vertebrates. Int. J. Leg. Med..

[bib27] Kniha E., Aspöck H., Auer H., Walochnik J. (2023). *Leishmania* infections and *Leishmania* species in central Europe. Wiener Tierärztliche Monatsschrift - Vet Med Austria.

[bib28] Kniha E., Dvořák V., Halada P., Milchram M., Obwaller A.G., Kuhls K. (2020). Integrative approach to *Phlebotomus mascittii* Grassi, 1908: First record in Vienna with new morphological and molecular insights. Pathogens.

[bib29] Kniha E., Milchram M., Dvořák V., Halada P., Obwaller A.G., Poeppl W. (2021). Ecology, seasonality and host preferences of Austrian *Phlebotomus* (*Transphlebotomus*) *mascittii* Grassi, 1908, populations. Parasites Vectors.

[bib30] Koch L.K., Kochmann J., Klimpel S., Cunze S. (2017). Modeling the climatic suitability of leishmaniasis vector species in Europe. Sci. Rep..

[bib31] Larkin M.A., Blackshields G., Brown N.P., Chenna R., McGettigan P.A., McWilliam H. (2007). Clustal W and Clustal X version 2.0. Bioinformatics.

[bib32] Lee J.C.-I., Tsai L.-C., Huang M.-T., Jhuang J.-A., Yao C.-T., Chin S.-C. (2008). A novel strategy for avian species identification by cytochrome *b* gene. Electrophoresis.

[bib33] Lewis D.J. (1982). A taxonomic review of the genus *Phlebotomus* (Diptera: Psychodidae). Bull. Br. Museum.

[bib34] Maroli M., Feliciangeli M.D., Bichaud L., Charrel R.N., Gradoni L. (2013). Phlebotomine sandflies and the spreading of leishmaniases and other diseases of public health concern. Med. Vet. Entomol..

[bib35] Mary C., Faraut F., Lascombe L., Dumon H. (2004). Quantification of *Leishmania infantum* DNA by a real-time PCR assay with high sensitivity. J. Clin. Microbiol..

[bib36] Matsuno K., Weisend C., Kajihara M., Matysiak C., Williamson B.N., Simuunza M. (2015). Comprehensive molecular detection of tick-borne phleboviruses leads to the retrospective identification of taxonomically unassigned bunyaviruses and the discovery of a novel member of the genus *Phlebovirus*. J. Virol..

[bib37] Medlock J.M., Hansford K.M., Van Bortel W., Zeller H., Alten B. (2014). A summary of the evidence for the change in European distribution of phlebotomine sand flies (Diptera: Psychodidae) of public health importance. J. Vector Ecol..

[bib38] Mihalca A.D., Cazan C.D., Sulesco T., Dumitrache M.O. (2019). A historical review on vector distribution and epidemiology of human and animal leishmanioses in Eastern Europe. Res. Vet. Sci..

[bib39] Minnick M.F., Anderson B.E., Lima A., Battisti J.M., Lawyer P.G., Birtles R.J. (2014). Oroya fever and verruga Peruana: Bartonelloses unique to South America. PLoS Negl. Trop. Dis..

[bib40] Nicholas K.B., Nicholas H. (1997). GeneDoc: A tool for editing and annotating multiple sequence alignments. http://www.pscedu/biomed/genedoc.

[bib41] Obwaller A.G., Karakus M., Poeppl W., Töz S., Özbel Y., Aspöck H., Walochnik J. (2016). Could *Phlebotomus mascittii* play a role as a natural vector for *Leishmania infantum*? New data. Parasites Vectors.

[bib42] Oerther S., Jöst H., Heitmann A., Lühken R., Krüger A., Steinhausen I. (2020). Phlebotomine sand flies in Southwest Germany: An update with records in new locations. Parasites Vectors.

[bib43] Pedregosa F., Varoquaux G., Gramfort A., Michel V., Thirion B., Grisel O. (2011). Scikit-learn: Machine learning in Python. J. Mach. Learn. Res..

[bib44] Pérez-Ruiz M., Collao X., Navarro-Marí J.-M., Tenorio A. (2007). Reversetranscription, real-time PCR assay for detection of Toscana virus. J. Clin. Virol..

[bib45] Péterfi Z., Nemes Z., Vigvári S., Szomor Á., Kereskai L., Kucsera I. (2011). Visceral leishmaniasis in an immunocompetent Hungarian adult patient. Health.

[bib46] Prudhomme J., Delabarre A., Alten B., Berberoglu U., Berriatua E., Bongiorno G. (2024). Performance evaluation of nine reference centers and comparison of DNA extraction protocols for effective surveillance of *Leishmania*-infected phlebotomine sand flies: Basis for technical recommendations. PLoS Negl. Trop. Dis..

[bib47] Ready P.D. (2013). Biology of phlebotomine sand flies as vectors of disease agents. Annu. Rev. Entomol..

[bib48] Röhrig E., Hamel D., Pfister K. (2011). Retrospective evaluation of laboratory data on canine vector-borne infections from the years 2004–2008. Berl. Münchener Tierärztliche Wochenschr..

[bib49] Symeonidou I., Sioutas G., Gelasakis A.I., Tsokana C.N., Papadopoulos E. (2023). Leishmaniosis in Greece: The veterinary perspective. Pathogens.

[bib50] Tánczos B., Balogh N., Király L., Biksi I., Szeredi L., Gyurkovsky M. (2012). First record of autochthonous canine leishmaniasis in Hungary. Vector Borne Zoonotic Dis..

[bib51] Trájer A.J. (2017). Checklist, distribution maps, bibliography of the Hungarian *Phlebotomus* (Diptera: Psychodidae) fauna complementing with the climate profile of the recent sandfly distribution areas in Hungary. Folia Faun. Slovaca.

[bib52] Trájer A.J. (2021). The potential impact of climate change on the seasonality of *Phlebotomus neglectus*, the vector of visceral leishmaniasis in the East Mediterranean region. Int. J. Environ. Health Res..

[bib53] Trájer A.J., Bede-Fazekas A., Hufnagel L., Horvath L., Bobvos J., Paldy A. (2013). The effect of climate change on the potential distribution of the European *Phlebotomus* species. Appl. Ecol. Environ. Res..

[bib54] Trájer A.J., Kniha E. (2025). Climatic and meteorological factors shaping the potential activity season of sand flies in Southeast Europe. Acta Trop..

[bib55] Tsirigotakis N., Pavlou C., Christodoulou V., Dokianakis E., Kourouniotis C., Alten B., Antoniou M. (2018). Phlebotomine sand flies (Diptera: Psychodidae) in the Greek Aegean islands: Ecological approaches. Parasites Vectors.

[bib56] Vaselek S. (2021). Systematic review: Re-emergence of human leishmaniasis in the Balkans. Trop. Med. Int. Health.

[bib57] Vaselek S. (2021). Canine leishmaniasis in Balkan – a review of occurrence and epidemiology. Acta Trop..

[bib58] Vaselek S., Ayhan N., Oguz G., Erisoz Kasap O., Savić S., Di Muccio T. (2017). Sand fly and *Leishmania* spp. survey in Vojvodina (Serbia): First detection of *Leishmania infantum* DNA in sand flies and the first record of *Phlebotomus* (*Transphlebotomus*) *mascittii* Grassi, 1908. Parasites Vectors.

[bib59] Veiga J., Collantes F., Hernández-Triana L.M., Prosser S.W.J., Valera F. (2024). Multihost/Multivector community network: Disentangling sandfly species and host interactions in avian habitats. Transbound. Emerg. Dis..

[bib60] Velo E., Bongiorno G., Kadriaj P., Myrseli T., Crilly J., Lika A. (2017). The current status of phlebotomine sand flies in Albania and incrimination of *Phlebotomus neglectus* (Diptera, Psychodidae) as the main vector of *Leishmania infantum*. PLoS One.

[bib61] Wasserberg G., Yarom I., Warburg A. (2003). Seasonal abundance patterns of the sandfly *Phlebotomus papatasi* in climatically distinct foci of cutaneous leishmaniasis in Israeli deserts. Med. Vet. Entomol..

[bib62] Weidmann M., Sanchez-Seco M.P., Sall A.A., Ly P.O., Thiongane Y., Lô M.M. (2008). Rapid detection of important human pathogenic phleboviruses. J. Clin. Virol..

[bib63] WHO (2021). https://www.who.int/news-room/fact-sheets/detail/leishmaniasis.

[bib64] Xhekaj B., Hoxha I., Platzgummer K., Kniha E., Walochnik J., Sherifi K. (2023). First detection and molecular analysis of *Leishmania infantum* DNA in sand flies of Kosovo. Pathogens.

[bib65] Xhekaj B., Hoxha I., Platzgummer K., Stefanovska J., Dvořák V., Milchram M. (2024). A cross‐sectional study on phlebotomine sand flies in relation to disease transmission in the Republic of Kosovo. Med. Vet. Entomol..

