# Supplementary material for: Updating the distribution of sand flies in Hungary with implications on their biology and ecology
Source: Curr Res Parasitol Vector Borne Dis. 2025 Jul 8;8:100293. doi: 10.1016/j.crpvbd.2025.100293 (PMC12274776; doi:10.1016/j.crpvbd.2025.100293)
Supplement: Multimedia component 2 [file mmc2.pdf]

Supplementary Table S2. Primers and probes used in RT-PCR-based protocols for the detection of *Phlebovirus* RNA used in this study.

Target	Primers and probes	Sequence (5'-3')	Concentration (nM)	Reference
Pan-Phlebovirus (L segment)	TBPVL2759F	CAGCATGGIGGICTIAGAGAGAT	1600	Matsuno et al. (2015)
	TBPVL3267R	TGIAGIATSCCYTGCATCAT	1600	
	HRT-GL2759F	CAGCATGGIGGIYTIAGRGAAATYTATGT	1600	
	HRT-GL3276R	GAWGTRWARTGCAGGATICCYTGCATCAT	1600	
Toscana virus (S RNA, N gene)	STOS-F	TGCTTTTCTTGATGAGTCTGCAG	1000	Pérez-Ruiz et al. (2007)
	STOS-R	CAATGCGCTTYGGRTCAAA	1000	
	STOS-P	FAM-ATCAATGCATGGGTRAATGAGTTTGCTTACC-TAMRA	200	
	TOS F	GGGTGCATCATGGCTCTT	500	Weidmann et al. (2008)
	TOS R	GCAGRGACACCATCACTCTGTC	500	
	TOS P	FAM-CAATGGCATCCATAGTGGTCCCAGA-TAMRA	200	
	TOS-IMT-F	TCTCCCAGGAAATGACATCC	400	Brisbarre et al. (2015)
	TOS-IMT-R	AGATGGGWGTCTCTGGTCAT	400	
	TOS-IMT-P	FAM-TGTGGTYCAAGCAGCACGGGTG-TAMRA	200	
Sicilian virus (S RNA, N gene)	SFSV-All-F	ATGGASGASTACCAGAARATYGC	400	Alwassouf et al. (2016)
	Corfou-Toros-F	ATGGAGGACTACCAGAAGATCGC	400	
	Corfou-Toros-R	CTAGCATCAAAACCYTGGTAAGCAAA	400	
	SFSV-DAHV-R	CTGGCATCAAAYCCYTGATAIGCAAA	400	
	SFSV-DAHV-F	ATGGACGAGTACCAGAAAATTGC	400	
	Corfou-Toros-P	FAM-TTCGGTGAGCAGGCTATAGATGA-TAMRA	400	
	SFSV-P1	FAM-TTTGGAGAACAGGCCATTGATGAG-TAMRA	400	
	SFSV-P2	FAM-TTTGGAGAGCAGGCTATTGATGAG-TAMRA	400	
