# Supplementary material for: Updating the distribution of sand flies in Hungary with implications on their biology and ecology
Source: Curr Res Parasitol Vector Borne Dis. 2025 Jul 8;8:100293. doi: 10.1016/j.crpvbd.2025.100293 (PMC12274776; doi:10.1016/j.crpvbd.2025.100293)
Supplement: Multimedia component 3 [file mmc3.pdf]

Supplementary Table S3. The bioclimatic variables used in the linear discriminant analysis (LDA).

**Climatic variable**	**Abbreviation**	**Unit**
Annual Mean Temperature	bio1	°C
Mean Diurnal Range (Mean of monthly (max temp - min temp))	bio2	°C
Isothermality (bio2/bio7) (×100)	bio3	°C
Temperature Seasonality (standard deviation ×100)	bio4	°C
Max Temperature of Warmest Month	bio5	°C
Min Temperature of Coldest Month	bio6	°C
Temperature Annual Range (bio5-bio6)	bio7	°C
Mean Temperature of Wettest Quarter	bio8	°C
Mean Temperature of Driest Quarter	bio9	°C
Mean Temperature of Warmest Quarter	bio10	°C
Mean Temperature of Coldest Quarter	bio11	°C
Annual Precipitation	bio12	mm
Precipitation of Wettest Month	bio13	mm
Precipitation of Driest Month	bio14	mm
Precipitation Seasonality (Coefficient of Variation)	bio15	mm
Precipitation of Wettest Quarter	bio16	mm
Precipitation of Driest Quarter	bio17	mm
Precipitation of Warmest Quarter	bio18	mm
Precipitation of Coldest Quarter	bio19	mm
