# Supplementary material for: Updating the distribution of sand flies in Hungary with implications on their biology and ecology
Source: Curr Res Parasitol Vector Borne Dis. 2025 Jul 8;8:100293. doi: 10.1016/j.crpvbd.2025.100293 (PMC12274776; doi:10.1016/j.crpvbd.2025.100293)
Supplement: Multimedia component 4 [file mmc4.pdf]

Supplementary Table S4. Occurrence by number of positive locations of sand fly species based on land cover.

		Ph. mascittii			*Ph. neglectus*			*Ph. papatasi*		
		**Peri-urban**	**Agricultural**	**Rural**	**Peri-urban**	**Agricultural**	**Aural**	**Peri-urban**	**Agricultural**	**Rural**
Ph. mascittii	**Peri-urban**	8	-	-	-	-	-	1	-	-
	**Agricultural**	-	-	-	-	-	-	-	-	-
	**Rural**	-	-	1	-	-	2	-	-	-
Ph. neglectus	**Peri-urban**				1	-	-	1	-	-
	**Agricultural**				-	-	-	-	-	-
	**Rural**				-	-	2	-	-	-
Ph. papatasi	**Peri-urban**							1	-	-
	**Agricultural**							-	-	-
	**Rural**							-	-	-
